# Paeoniflorin Protects against Ischemia-Induced Brain Damages in Rats via Inhibiting MAPKs/NF-κB-Mediated Inflammatory Responses

**DOI:** 10.1371/journal.pone.0049701

**Published:** 2012-11-14

**Authors:** Ruo-Bing Guo, Guo-Feng Wang, An-Peng Zhao, Jun Gu, Xiu-Lan Sun, Gang Hu

**Affiliations:** 1 Jiangsu Key Laboratory of Neurodegeneration, Department of Pharmacology, Nanjing Medical University, Nanjing, China; 2 Department of Cadre Ward No. 3, the General Hospital of Jinan Military Area Command of PLA, Jinan, China; University of South Florida, United States of America

## Abstract

Paeoniflorin (PF), the principal component of Paeoniae Radix prescribed in traditional Chinese medicine, has been reported to exhibit many pharmacological effects including protection against ischemic injury. However, the mechanisms underlying the protective effects of PF on cerebral ischemia are still under investigation. The present study showed that PF treatment for 14 days could significantly inhibit transient middle cerebral artery occlusion (MCAO)-induced over-activation of astrocytes and microglia, and prevented up-regulations of pro-inflamamtory mediators (TNFα, IL-1β, iNOS, COX_2_ and 5-LOX) in plasma and brain. Further study demonstrated that chronic treatment with PF suppressed the activations of JNK and p38 MAPK, but enhanced ERK activation. And PF could reverse ischemia-induced activation of NF-κB signaling pathway. Moreover, our *in vitro* study revealed that PF treatment protected against TNFα-induced cell apoptosis and neuronal loss. Taken together, the present study demonstrates that PF produces a delayed protection in the ischemia-injured rats via inhibiting MAPKs/NF-κB mediated peripheral and cerebral inflammatory response. Our study reveals that PF might be a potential neuroprotective agent for stroke.

## Introduction

Stroke has a high incidence and is harmful to human health. It is a leading cause of death and the third cause of disability. The loss of neurological functions following stroke is caused by massive loss of neurons resulting from hypoxic-ischemic insults. Up to now, several hypotheses have been put forward, such as Ca^2+^ overload, oxidative injury, excitotoxicity and apoptosis [1]. A complex interplay between different factors and signal cascades results in neuronal cell degeneration after ischemia [2]–[4]. Extensive loss of neurons as well as glia over-activations in ischemic brain are the characteristic pathological features of cerebral ischemia [5]. The acute neuronal damage is followed by a second round of neuronal injury that occurs hours to days after brain ischemia in the neighboring areas, which is called delayed neuronal death (DND) [6]. It is very important to study the mechanism underlying DND for prolonging the timescale of clinical treatment [7].

Much progress has been made in developing novel therapeutics to treat stroke, including glutamate receptor antagonists, calcium channel blockers, radical scavengers, and anti-apoptotic agents [8]. Despite many promising neuroprotective agents have been identified in extensive animal research, few has been translated into clinically effective therapies [9]. Tissue plasminogen activators (tPAs) are still the only agents approved by the Food and Drug Administration (FDA). tPAs has limited applicability and is currently used in fewer than 5% of stroke victims [10], although there is now some evidence that the window of opportunity for tPA usage may be extended to 6 h after the event [11]; [12]. Therefore, arduous works should be directed at increasing efficacy and extending the treatment window for stroke.

Given the complex pathophysiology of stroke, it may be unrealistic to hope for a single “magic bullet” that will result in neuroprotection and rescue of damaged but not-yet-destroyed neurons [13]. To date, no single agent has been shown to improve stroke outcome in humans, and the current standard of care remains primarily supportive. The herb Paeonia lactiflora pall, which is known as “Shao Yao” in Chinese, has been used for more than 1,000 years in traditional Chinese medicine to treat cramp, pain, giddiness and congestion [14]. Paeoniflorin (PF), a monoterpene glucoside, is the principal bioactive component purified and extracted from the root of Paeonia lactiflora pall. It has been reported to exhibit many pharmacological effects such as anti-inflammation, anti-allergy, anti-hyperglycemia, analgesia [15], blocking neuromusculus [16] and enhancing cognition [17]; [18]. Previous studies indicated the protective effects of PF may be related to its abilities to prevent apoptosis [14], scavenge free radicals [19], adjust cerebral energy metabolism and nitric oxide formation [20], prevent thrombosis [21], block Na^+^ channels, activate adenosine A1 receptor [22]; [23] or facilitate the translocation of protein kinase C and glucose transporter [24]. These studies suggest that multitargets may be involved in PF-mediated protective effects. Regarding to the effects of PF in the cerebral ischemia, it has been reported that treatment with PF attenuated ischemia-induced pathological and behavioral changes as well as cognitive impairments [18]; [23]; [25]–[27]. However, the mechanisms underlying the protective effects of PF on cerebral ischemia are still under investigation. Therefore, the present study focused on evaluating the delayed protective effects of PF in the transient middle cerebral artery occlusion (MCAO) rat model, and revealing the signaling pathways involved in the actions of PF.

## Experimental Procedures

### 1. Animals

Male SD rats weighing 220–250 g were used in the present study. Animals were allowed to acclimatize for at least 7 days prior to experimentation. The animals were housed in individual cages under light-controlled conditions and at room temperature. Food and water were available *ad libitum*. All animals received care in compliance with the Guide for the Care and Use of Laboratory Animals published by the National Institutes of Health (NIH publication 80–23, revised 1996). The rats were randomly divided into the following 3 groups (*n* = 20–25 for each group): 1. MCAO groups: MCAO(90 min)+saline (2 ml.kg^−1^, *i.p.*, twice per day for 14 days); 2. PF groups: PF (5 mg. kg^−1^, *i.p.*, twice per day) was administrated for 14 days after MCAO (90 min) and reperfusion 24 h. PF (purity>98.5%) was purchased from Nanjing ZeLang Medical Technology Co., LTD); 3. Sham group. Physiological parameters of rats were monitored continuously, and the temperature remains 37±1°C throughout the intraoperative period.

### 2. Establishment of transient MCAO

The experimental MCAO rat model was conducted as described previously (Takano *et al.*, 1997), with minor modifications. Briefly, the rats were anesthetized with choral hydrate (300 mg. kg^−1^, *i.p.*), the bifurcation of the left common carotid artery was exposed. The right MCA was occluded for 90 min by insertion of a monofilament nylon suture through the common carotid artery as described previously. Then, the suture was withdrawn allowing reperfusion.

### 3. Determination of neurological symptoms

The severity of neurological symptoms of the experimental animals was graded on a scale of 0′–5′ according to methods described previously with slight modifications as follows: 0-no neurological deficit; 1′-retracts left forepaw when lifted by the tail; 2′-circles to the left; 3′-falls while walking; 4′-does not walk spontaneously; 5′-dead. Neurological symptoms were evaluated 24 h after MCAO. The above behavioral observations were carried out in a blinded manner.

### 4. Measurement of infarct size

Coronal sections of the brain were cut into 2 mm slices and immersed in 2% 2,3,5-triphenyltetrazolium chloride (TTC; Sigma, USA) at 37°C for 15 min, followed by 10% formaldehyde solution. The image of each slice was captured by digital camera. The infarct area and hemisphere area were traced and quantitated by an image analysis system (Adobe ImageReady 7.0). Analysis of cerebral ischemic damage included total (hemispheric), cortical and subcortical (striatal) infarction.

### 5. Immunohistochemistry

For immunohistochemical analysis, rats were perfused with saline followed by 4% paraformaldehyde and immunohistochemistry was performed on 40 µm free-floating sections using anti-NeuN monoclonal antibody (1∶100, Millipore), anti-GFAP rabbit monoclonal antibody (1∶400, Millipore), or CD11b monoclonal antibody (1∶400, Abcam). Sections were then incubated with corresponding secondary antibodies and immunoreactivity was visualized with 0.05% DAB as chromagen. Negative controls received the same treatments omitting the primary antibodies and showed no specific staining.

### 6. Western blot analysis

The isolated cortex, hippocampus and striatum were homogenized in lysis buffer (20 mM Tris pH 7.5, 150 mM NaCl, 1% nonidet P-40, 0.5% sodium deoxycholate and protease inhibitor cocktail), centrifuged at 3,000 g for 10 min. Protein concentration was determined by BioRad protein assay. Samples were electrophoresed in SDS/PAGE gels and transferred onto a PVDF membrane. The membrane was blocked with 5% nonfat milk in 1×TBS, 0.1% Tween-20 at 25°C for 1 h and subsequently incubated overnight at 4°C with appropriate primary antibody (anti-iNOS, anti-COX2, anti-5-LOX, anti-phospho-p38 MAPK, anti-phospho-ERK, anti-phospho-JNK, anti-NF-κB p65, anti-IκBα, anti-cytochrome *c*, anti-Bcl-2 and anti-Bax were purchased from Santa Cruz, Abcam or Cell Signaling) diluted in TBST [TBS, 0.1% (v/v) Tween-20 and 5% (w/v) BSA]. After incubated with horseradish peroxidase-conjugated secondary antibodies for 1 h, the blots were developed with chemiluminescence reagent.

### 7. Measurement of plasma TNFα and IL-1β levels

Plasma TNFα and IL-1β levels were measured using enzyme-linked immunosorbent assay (ELISA) kit (purchased from ExCell) according to manufacturer's recommendations.

### 8. Measurement of mRNA levels of TNFα and IL-1β in the brain

Total RNA was extracted using Trizol reagent (Invitrogen Life technologies, USA) followed by treatment with RNase-free DNaseI (Invitrogen Life technologies, USA). Reverse transcription was performed with the One-Step RNA-PCR Kit (Takara), according to the manufacturer's protocol. PCR primers were as follows: glyceraldehyde-3-phosphate dehydrogenase (GAPDH) was used as housekeeping gene (forward 5′- CCTACCCCCAATGTATCCGTTGTG-3′ and reverse 5′- GGAGGAATGGGAGTTGCTGTTGAA-3′), TNF-α (forward 5′- CGAGTGACAAGCCCGTAG-3′ and reverse 5′- GGATGAACACGCCAGTCG-3′), IL1β (forward 5′- CCAGGATGAGGACCCAAGCA-3′ and reverse 5′- TCCCGACCATTGCTGTTTCC-3′). Quantitative real-time PCR was performed on a 7300 Real-Time PCR System using the SYBR Green PCR Master Mix. The samples were run in triplicate and the experiments were repeated at least three times. The GAPDH gene was used as an endogenous control to normalize for differences in the amount of total RNA in each sample. All values were expressed as fold increase or decrease relative to the expression of GAPDH.

### 9. Hippocampal primary neuron cultures

Primary neuron cultures were prepared from the hippocampal tissues of embryonic day 14/15 mice. Briefly cell were dissociated by trypsinization [0.25% (w/v) Trypsin and 0.02% EDTA in Ca^2+^ and Mg^2+^ free Hanks' balanced salt solution] at 37°C for 10 min, followed by gentle triturating in plating medium (h-DMEM supplemented with 10% fetal bovine serum and 10% horse serum, Gibco-BRL). Cells were seeded onto poly-L-lysine (Sigma) -coated 24-well plates and 25-cm^2^ T-flask at a density of 2.5×10^5^ cells per cm^2^ and incubated at 37°C in 5% CO_2_ atmosphere. After 18–24 h, after cells were adherenced, the medium was replaced by Neurobasal medium supplemented with 2% B-27 (Gibco-BRL) and 0.5 mM L-glutamine (Sigma) and treated with cytosine arabinoside (1 µM) for 24 h to inhibit glial cell proliferation. Half of the culture medium was replaced every 3.5 days. Cultures were used after 7 days in vitro.

### 10. Analysis of cell viability

Cell viability was determined by use of an MTT assay. Cells were seeded in 96-well plates at 1×10^4^ cells per well and grown to 70% confluence in culture medium. The medium was replaced by medium containing TNFα (100 µg/L) or TNFα+PF(25,50, 100 µM) for 24 h. A total of 5 g/L MTT was added to each well after 24 h, and the culture continued to incubate for another 4 h at 37°C. Then the medium was aspirated, dye crystals were dissolved in DMSO and the absorbance was read on an ELISA plate reader using a 490 nm filter.

### 11. Hoechst 33342 staining

After treatment with TNFα (100 µg/L) or TNFα+PF(50 µM) for 24 h, the primary cultured neurons were fixed with 4% paraformaldyhyde for 30 min at 25°C, then washed with pre-chilled phosphate buffer saline (PBS) three times and exposed to 10 mg/L Hoechst 33342 at room temperature in the dark for 10 min. Samples were observed under a fluorescence microscopy (Nikon Optical TE2000-S).

### 12. Statistics

For statistical analyses, a standard software package (SPSS for Windows 10.1) was used. All data were given as means ± SD. Differences between groups were compared by using a one-way analysis of variance (ANOVA). *P* values<0.05 were considered significant.

## Results

### 1. PF treatment improves neurological deficits and decreases cerebral infarct size in rats

**Figure 1:**
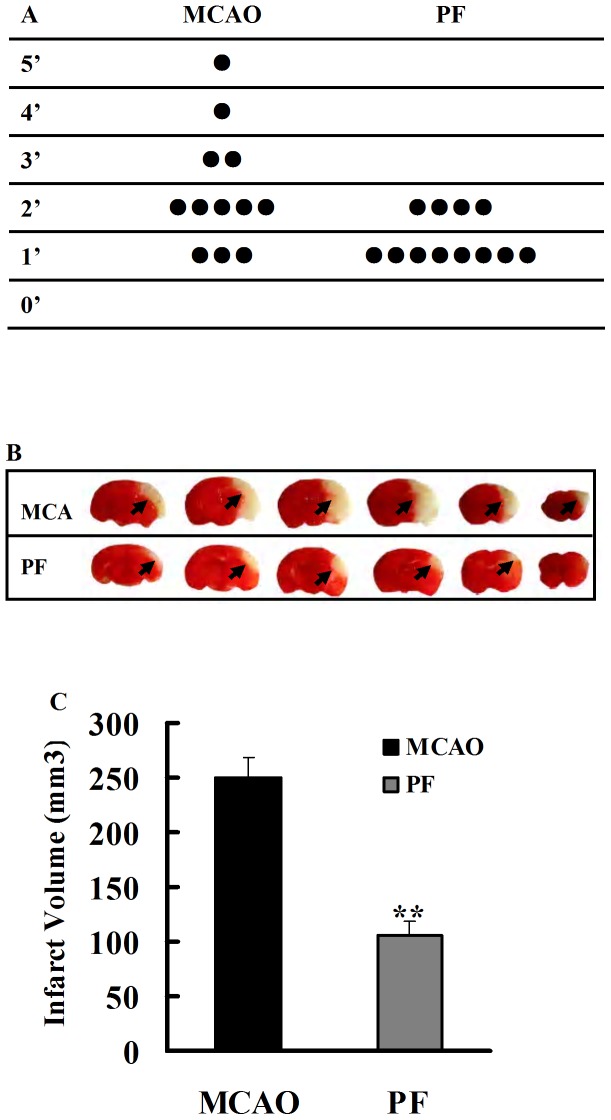
Effects of PF on the size of cerebral infarct and the neurological deficits. (A) The neurological score of MCAO groups and PF groups (n = 12). (B) Representative photographs showing the cerebral infarct of rat brain slices measured by 2, 3, 5-triphenyltetrazolium chloride (TTC) staining(n = 8). Black arrow indicated “infarcted area”. (C) The infarct volume of MCAO groups and PF groups. MCAO, rats treated with saline for 14 days after transient MCAO; PF, PF (5 mg. kg^−1^) was administered for 14 days after transient MCAO. All data were expressed as mean ± SD. **P<0.01 *vs* MCAO.

Rats treated with vehicle showed neurological deficits such as retracting left forepaw when lifted by the tail, circling to the left or falling while walking. PF treatment significantly improved the neurological symptoms (Figure 1A). The neurological deficit score and the infarct volume of rats were determined to evaluate the impacts of PF treatment for 14 days after MCAO. TTC staining analysis revealed a mean infarct volume of 250.1±17.2% mm^3^ in vehicle treated groups. Treatment with PF for 14 days significantly reduced the magnitude of ischemic lesion to 106.0±11.7 mm^3^ (*P*<0.01, Figure 1B and 1C). In addition, the neuroprotection of PF treatment were not to be accounted for by the modification of physiological variables, since the parameters (e.g. blood pH, pO2, pCO2 and blood glucose) were kept within normal physiologic limit (Table 1).

**Table 1:** Physiologic parameters of rats before, during MCAO or 15 min after saline and PF treatment.

Parameters	Monitored time	MCAO groups	PF groups
	before ischemia	7.35±0.02	7.31±0.02
**pH**	during occlusion	7.34±0.02	7.32±0.02
	15 minutes after reperfusion	7.37±0.02	7.31±0.01
	before ischemia	44. 8±2.5	46. 8±2.6
**pCO_2_ (mm Hg)**	during occlusion	42. 3±1.6	44. 8±3.8
	15 minutes after reperfusion	47. 4±3.1	47. 8±1.5
	before ischemia	85. 5±2.1	83. 7±2.4
**pO_2_(mm Hg)**	during occlusion	42. 3±1.6	44. 8±3.8
	15 minutes after reperfusion	81. 0±2.2	85. 5±1.9
	before ischemia	11. 0±1.2	11. 5±1.9
**Blood glucose (mmol/L)**	during occlusion	11. 5±0.4	11. 8±0.6
	15 minutes after reperfusion	10. 8±0.9	11. 3±0.5

Physiologic data obtained from control and drug-treated groups are presented as mean ± SD. All animals were maintained at 37±1°C. There were no statistically differences within or between the groups at any time point.

### 2. PF treatment prevents ischemia-induced loss of neuron, and inhibits activations of microglia and astrocytes in the brain

**Figure 2:**
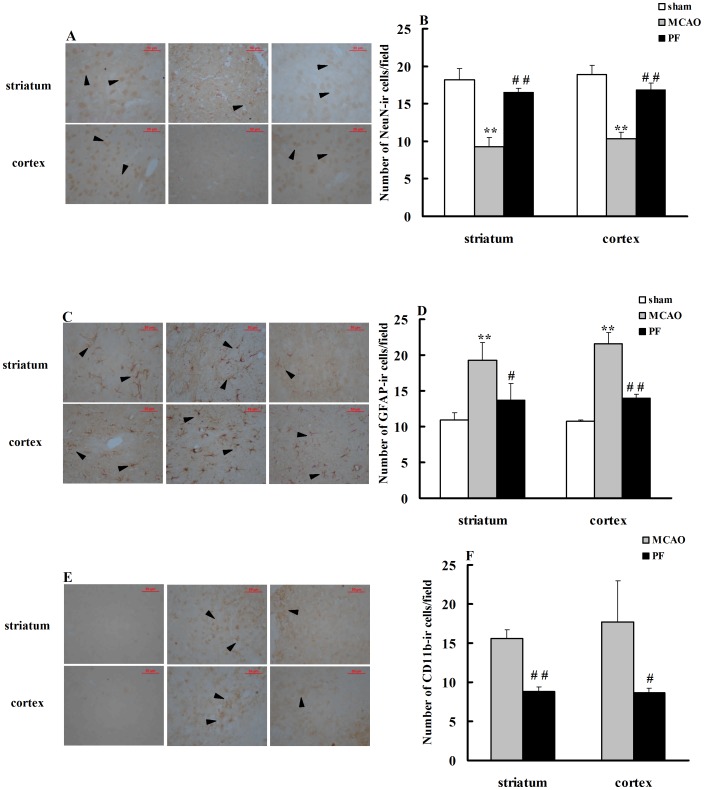
PF treatment inhibits activations of astrocytes and microglia, and prevents the loss of neuron. (A) Microphotographs of NeuN-ir cells in the cortex and striatum of rats with ×40 objective. (B) Stereological counts of NeuN-ir cells in the rat brain. Sham, rats received surgery without vessel occlusion. (C) Microphotographs of GFAP-ir cells in the cortex and striatum of rats with ×40 objective. (D) Stereological counts of GFAP-ir cells in the rat brain. (E) Microphotographs of CD11b-ir cells in the cortex and striatum of rats with ×40 objective. (F) Stereological counts of CD11b-ir cells in the rat brain. Sham, rats received surgery without vessel occlusion; MCAO, rats treated with saline for 14 days after transient MCAO; PF, PF (5 mg. kg^−1^) was administered for 14 days after transient MCAO. n = 4–6. All data were expressed as mean ± SD. Black arrows indicated the positive staining. * *P*<0.05,** *P*<0.01 vs sham; #*P*<0.05, ##*P*<0.01 vs MCAO. Scale bar, 50 µm.

As shown in Figure 2A and 2B, there was a robust loss of neurons in ipsilateral striatum and cortex after ischemia. However, neuronal injury in striatum and cortex was significantly prevented by PF treatment.

After ischemia, astrocytes displayed reactive changes in cortex and striatum of vehicle treated groups which are characterized by increasing the expression of glial fibrillary acidic protein (GFAP) and the number of GFAP-positive cells. The number of astrocytes of MCAO-treated groups was higher than that of sham groups. PF treatment significantly inhibited the astrocytic activation in the brain (Figure 2C and 2D).

Ischemia also induced significantly activation of microglia which was characterized by changing the resting ramified types to the fully amoeboid morphology. The number of activated microglia and the soma volume of microglia were markedly increased induced by ischemia, which were inhibited by treatment with PF (Figure 2E and 2F).

### 3. PF treatment decreases the levels of TNFα and IL-1β in plasma, down-regulates the protein expression levels of iNOS and COX_2_ and 5-LOX in the brain

**Figure 3:**
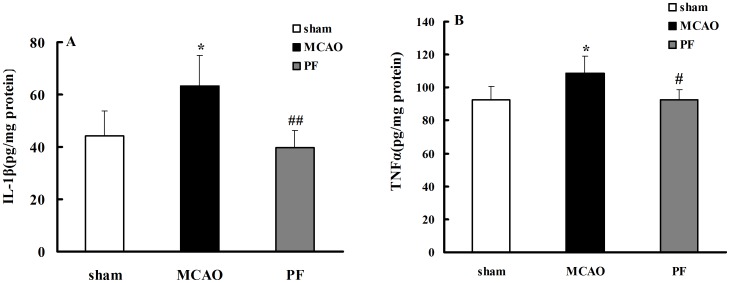
PF treatment decreases the levels of IL-1β(A) and TNFα(B) in plasma. Sham, rats received surgery without vessel occlusion; MCAO, rats treated with saline for 14 days after transient MCAO; PF, PF (5 mg. kg^−1^) was administered after MCAO for 14 days. n = 9. All data were expressed as mean ± SD. * *P*<0.05,** *P*<0.01 vs sham; #*P*<0.05, ##*P*<0.01 vs MCAO.

Ischemia and reperfusion resulted in 1.4-fold elevation in the concentrations of plasma interleukin 1β (IL-1β) and 1.2-fold elevation in the concentrations of plasma tumor necrosis factor α (TNFα). Treatment with PF markedly decreased the IL-1β level to 39.7, and the TNFα level to 92.5, respectively (Figure 3).

**Figure 4:**
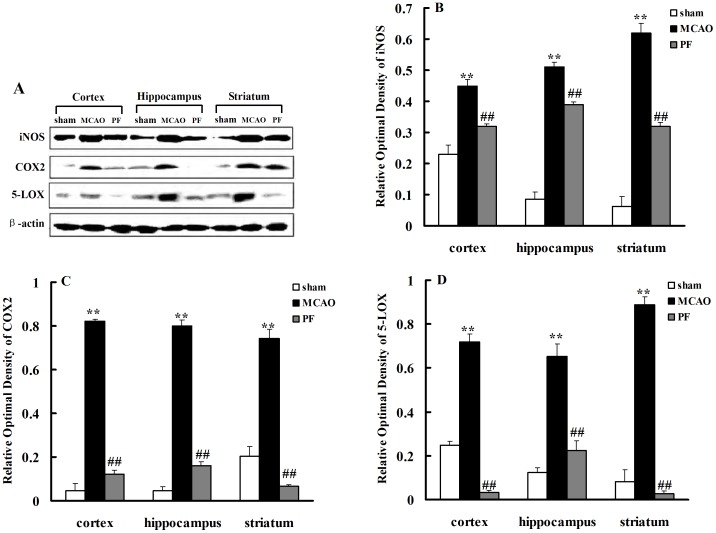
PF treatment inhibits the protein expressions of iNOS, COX-2 and 5-LOX in the brain. (A) represents the protein expression of iNOS, COX-2 and 5-LOX in the cortex, hippocampus and striatum of rats. (B–D) indicates the relative optical density of these proteins expression. Sham, rats received surgery without vessel occlusion; MCAO, rats treated with saline for 14 days after transient MCAO; PF, PF (5 mg. kg^−1^) was administered for 14 days after MCAO. n = 4. All data were expressed as mean ± SD. ** *P*<0.01 vs sham; ^##^ *P*<0.01 vs MCAO.

Simultaneously, we determined the protein expressions of inducible nitric oxide synthase (iNOS), cyclooxygenase 2 (COX2) and 5-lipoxidase (5-LOX) in the cortex, hippocampus and striatum via western blotting. The results showed that the protein levels of iNOS, COX_2_ and 5-LOX in the brain of MCAO-injured groups were higher compared to those in the sham groups. PF treatment for 14 days significantly inhibited the three protein expressions in the brain (Figure 4).

### 4. PF treatment inhibited mRNA expressions of TNFαand IL-1β in the brain

**Figure 5:**
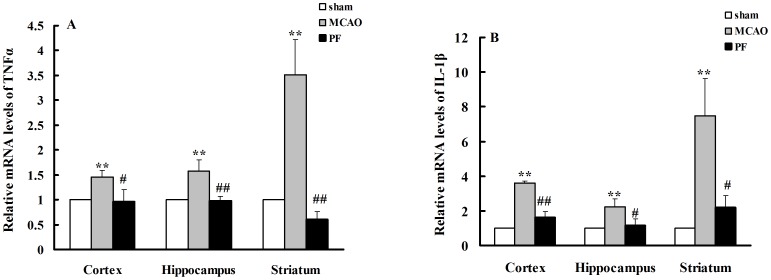
PF treatment decreases the mRNA expressions of TNFα (A) and IL-1β (B) in the brain. Sham, rats received surgery without vessel occlusion; MCAO, rats treated with saline for 14 days after transient MCAO; PF, PF (5 mg. kg^−1^) was administered for 14 days after MCAO; n = 4. All data were expressed as mean ± SD. ** *P*<0.01 vs sham, ^#^ *P*<0.05; ^##^ *P*<0.01 vs MCAO.

We further observed the changes in mRNA levels of IL-1β and TNFα in brain by real-time PCR analysis. Our results showed that ischemia attack significantly up-regulated the mRNA levels of TNFα to 145%, 158% and 352% in cortex, hippocampus and striatum, respectively. The mRNA levels of IL-1β in the three brain regions were also robustly increased by ischemic injury to 359%, 223% and 747% in cortex, hippocampus and striatum, respectively. PF treatment reversed the increase of mRNA levels of TNFα (Figure 5A), and remarkably decreased the mRNA levels of IL-1β in cortex, hippocampus and striatum of rats (Figure 5B).

### 5. PF treatment regulates MAPK and NF-κB signalling pathway

**Figure 6:**
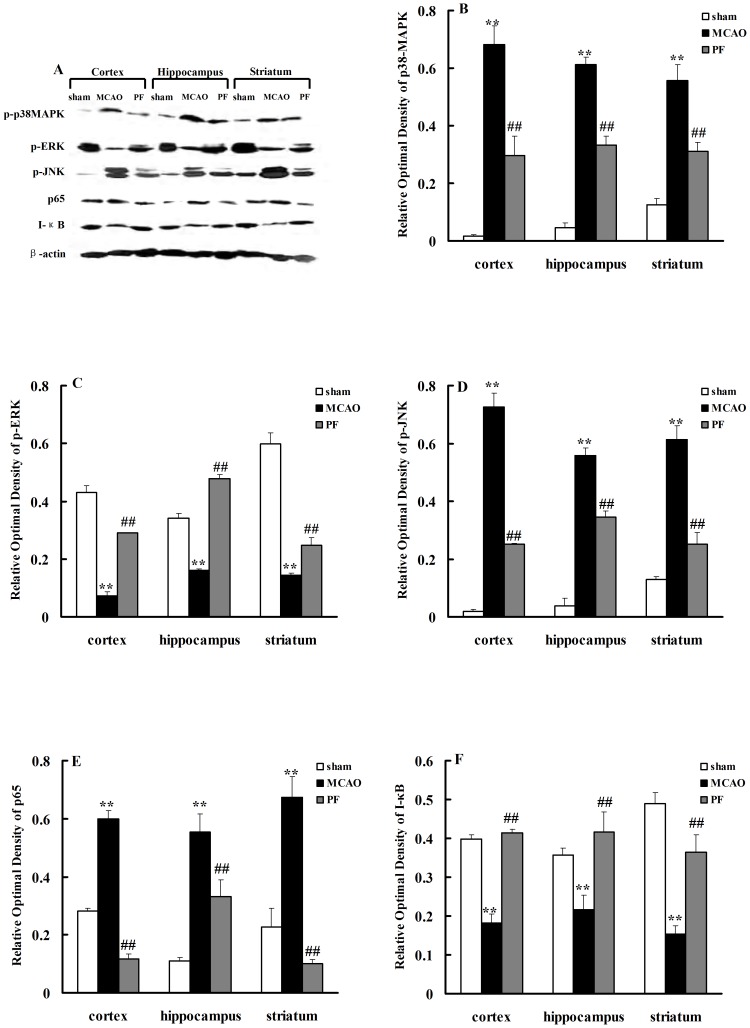
Effects of PF on MAPK and NF-κB signaling effectors expression. p-P38 MAPK, p-ERK, p-JNK, p65 and IκB protein expressions in the cortex, hippocampus and striatum of rats were indicated in (A). The relative optical densities were indicated in (B–F). Sham, rats received surgery without vessel occlusion; MCAO, rats treated with saline for 14 days after transient MCAO; PF, PF (5 mg. kg^−1^) was administered for 14 days after MCAO. n = 4. All data were expressed as mean ± SD. ** *P*<0.01 vs sham; ^##^ *P*<0.01 vs MCAO.

Since MAPK signaling pathway plays important roles in inflammatory response, the effects of PF treatment on mitogen activated protein kinases (MAPKs) signaling pathways including extracellular signal-regulated kinase (ERK), Jun N-terminal kinase (JNK) as well as p38 MAPK were explored. Ischemia and reperfusion up-regulated the phosphorylations of JNK and p38 MAPK, but down-regulated ERK phosphorylation. Treatment with PF significantly suppressed the activations of JNK(Figure 6D) and p38 MAPK(Figure 6B), but enhanced ERK activation (Figure 6C).

We further observed the ischemic injury-induced changes in the nuclear factor kappa B (NF-kB) signaling pathway. As shown in figure 6E and 6F, the expression of p65 was increased but ikappaB-alpha (I-κBα) was significantly decreased by ischemic insult. These changes in pathway were reversed by PF treatment.

### 6. PF treatment down-regulates the expressions of cytochrome c and Bax, but up-reglulates the expressions of Bcl-2

**Figure 7:**
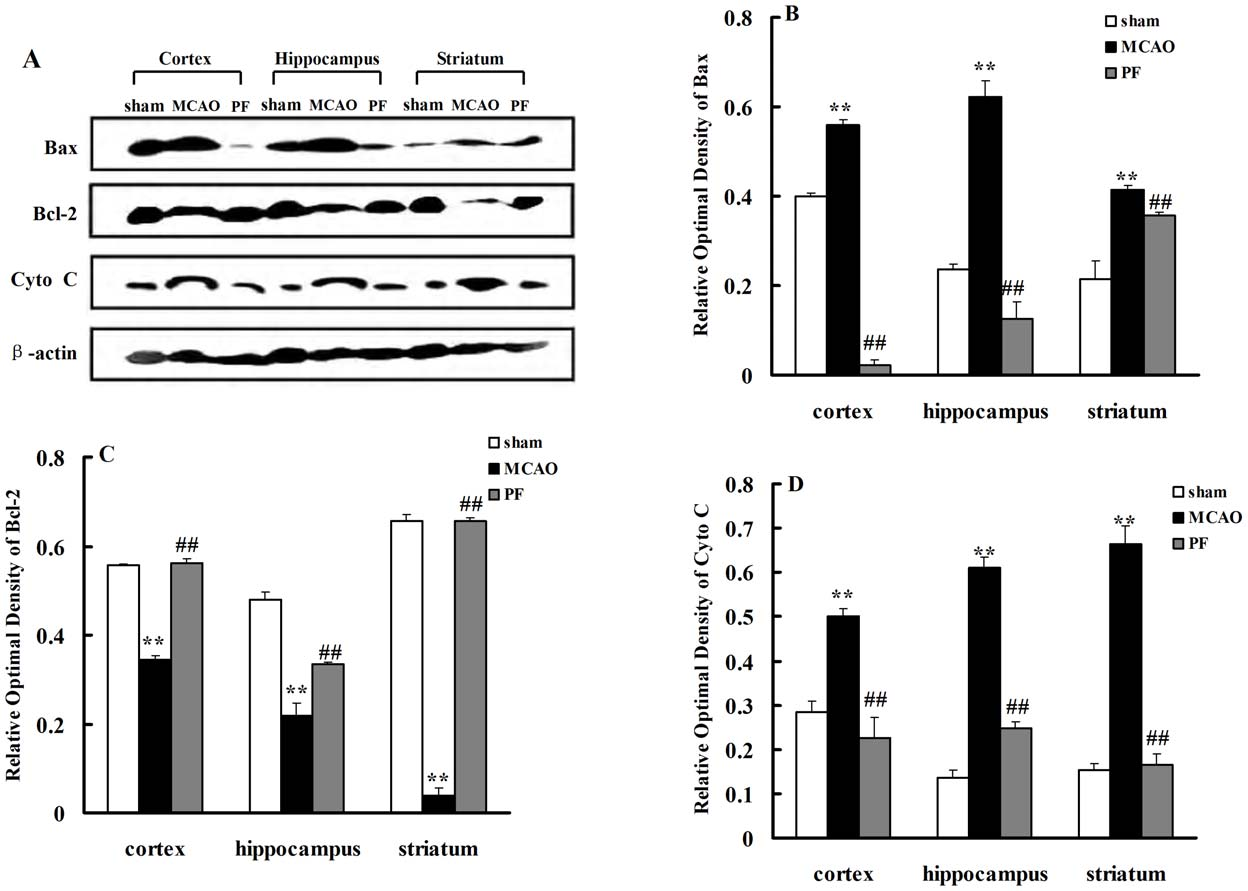
PF treatment decreases expressions of cytochrome c and Bax, but increases the expressions of Bcl-2. Cytochrome c, Bax and Bcl-2 protein expressions in the cortex, hippocampus and striatum of rats were indicated in (A). The relative optical densities were indicated in (B–D). Sham, rats received surgery without vessel occlusion; MCAO, rats treated with saline for 14 days after transient MCAO; PF, PF (5 mg. kg^−1^) was administered for 14 days after MCAO. n = 4. All data were expressed as mean ± SD. ** *P*<0.01 vs sham; ^##^ *P*<0.01 vs MCAO.

The pro-apoptotic protein Bax and the anti-apoptotic protein Bcl-2 are crucial determinants of the apoptotic response and also control the release of cytochrome c. To address whether PF treatment influences the expression of apoptosis-related proteins participated in ischemia and reperfusion. Western blot analysis revealed a significant increases in the protein levels of cytoplasmic cytochrome c and Bax in the cortex, hippocampus and striatum, whereas there was a marked decrease in Bcl-2 expression (Figure 7), indicating higher apoptosis existed in the ischemic brain. Chronic treatment with PF could prevent the increases of Bax and cytochrome *c* (Figure 7B and 7D) and significantly elevated the protein levels of Bcl-2 (Figure 7C).

### 7. PF treatment protects TNFα-induced cytotoxicity in hippocampal neurons

**Figure 8:**
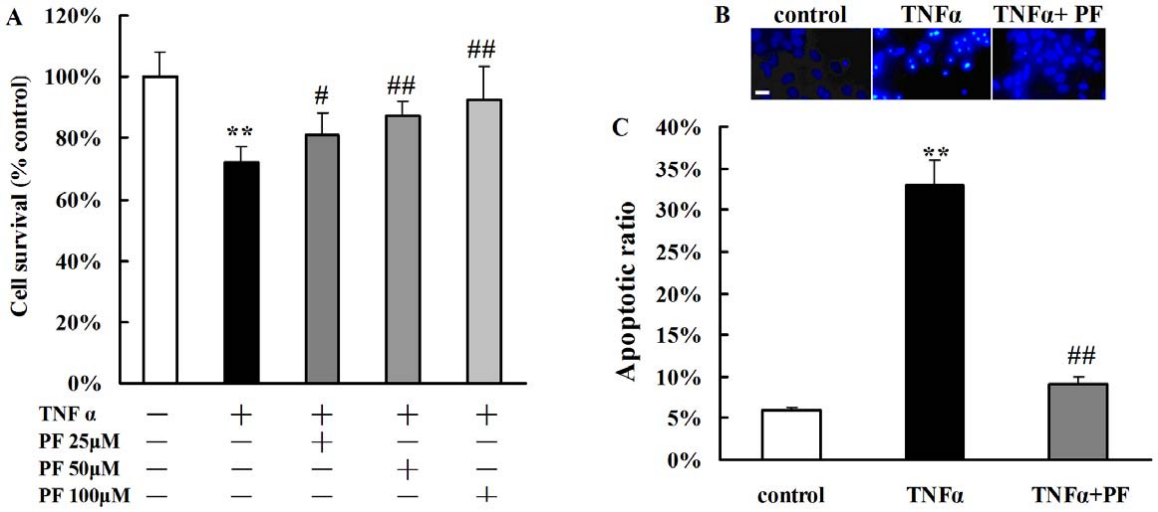
PF treatment protects TNFα-induced cytotoxicity in hippocampal neurons as assessed by MTT(A) and staining with Hoechst33342 (B and C). A and C. TNFα stimulation increased cell apoptosis and cell death in hippocampal neurons. PF inhibited the apoptotic ratio of neurons and promoted cell survival. B. Fluorescence photomicrographs of neurons with Hoechst 33242 staining. Bar = 50 µm. **P<0.01 *vs*. control group, ^#^ *P*<0.05, ^##^ *P*<0.01 *vs*. TNFα group. Data are means ± S.E.M. n = 4.

Many studies have been revealed that TNFα could activate both MAPKs and NF-kB signal pathways. So we used TNFα to induce inflammatory damage in hippocampal neurons, and to verify whether MAPKs and NF-kB signal pathways were involved in the neuroprotective effects of PF. After incubation with TNFα, the cell viability was decreased to 72.1%. Treatment with PF (25, 50, 100 µM) decreased the cell death rate in a concentration-dependent manner (cell survival ratio was 81.2%, 87.1% and 92.3%, respectively. Figure 8A).

The Hoechst 33342 staining that is sensitive to DNA was used to assess changes in nuclear morphology following TNFα or TNFα+PF treatment. The nuclei in normal cells were normal and exhibited diffused staining of the chromatin. However, after exposure to TNFα 100 µg/L for 24 h, neurons underwent typical morphologic changes of apoptosis such as condensed chromatin and shrunken nucleus. Treatment with PF (50 µM) almost reversed TNFα-induced neuronal apoptosis (Figure 8B and 8C).

## Discussion

An important delayed mechanism beginning within hours from the onset of ischemia is the robust inflammatory response in the ischemic tissue [5]; [28]; [29]. There is increasing evidence showing a detrimental effect of the post-ischemic inflammatory reaction. Therefore, therapeutic strategies targeting the delayed inflammatory response could inhibit the progression of the tissue damage providing an extended therapeutic window for neuroprotection.

Within hours after the onset of focal cerebral ischemia, peripheral leukocytes (granulocytes, monocytes/macrophages, lymphocytes) adhere to the cerebral endothelium, cross the vessel wall and invade the damaged parenchyma [28]; [30]. Activation and accumulation of leukocytes results in further damage. Current evidence suggests a detrimental role of iNOS and COX_2_ from neutrophils and vascular cells in the ischemic brain [31]. iNOS is transcriptionally induced in the ischemic core generating toxic levels of NO continuously [32]. Inhibition of iNOS by pharmacological and genetic approaches prevents ischemia-induced neurodegeneration. For example, iNOS null mice were reported to have smaller infarcts and better neurological outcomes after focal ischemia [33]. In the present study, ischemia elicited significantly increased expressions of iNOS, COX2 and 5-LOX in three brain regions, PF treatment inhibited the overexpressions of the three proteins. Simultaneously, we found that levels of TNFα and IL-1β in the plasma were also increased, PF treatment significantly down-regulated the levels of the two inflammatory factors.

In response to ischemic injury in brain, microglia and astrocytes are activated [34]. Microglia are the resident macrophages of the brain. They are very sensitive to subtle alterations in their neuronal microenvironment. The surrounding astrocytes are also sensitive to the increased release of these immunomodulatory peptides and therefore severe ischemia also compromises astrocytic function [28]; [34]. In response to the ischemic injury, glial cells quickly become activated and undergo morphological transformations, and are accompanied by functional changes, such as increasing expression of cytokines: interleukins (IL-1β, IL-4, IL-6, IL-10), TNFα, interferons and chemokines [35]; [36]. The accumulaiton of pro-inflammatory factors should further induce ischemic damages [35]; [37]; [38]. In the present study, we found ischemic insult resulted in over-activation of astrocytes and microglia, and thereby robustly elevated the mRNA expressions of TNFα and IL-1β in the brain. Chronic treatment with PF could inhibit glial over-activations, and reduce the mRNA levels of these inflamamtory factors. Moreover, our other study via comparative proteomics analysis showed that ischemic injury substantially increased the expressions of astrocyte marker proteins (S100β and GFAP), PF treatment significantly reversed these changes (data not published). Therefore, our results reveal that PF exerts the delay neuroprotective effects via inhibiting peripheral and cerebral inflammatory response.

The secretion of inflammatory molecules in cerebral ischemia triggers the activation of several transcription factors involved in the inflammatory response [39]. Among them, the activation of NF-kB and subsequent degradation of I-κBα are the key events in ischemia and reperfusion [40]. The activated NF-kB further induces the expression of genes encoding cell adhesion molecules and cytokines, thereby triggering the vicious cycle and exacerbating inflammatory injury [7]. Our results demonstrated that ischemia caused the overexpression of p65 and decreased expression of its endogenous inhibitor I-κBα, while these changes were reversed by PF treatment. Consequently, inhibiting NF-κB activation by PF resulted in the down-regulations of IL-1β and TNFα mRNA levels in the brain.

MAPKs are a family of key proteins which are involved in a wide range of cell responses, including cell proliferation, differentiation and apoptosis [41]. MAPK signaling pathways also positively regulate transcription of inflammatory genes, such as those coding for TNFa, IL-1β, and COX2 [42]. Both the JNK pathway and pro-inflammatory mediators further potentiate the brain tissue injury and lead to apoptotic and necrotic cell death of the potential viable tissue within hours and days [35]. Inhibition of MAP kinases (MAPK), especially p38 and JNKs, could lead to a reduction in pro-inflammatory molecule production by inflammatory cells, especially microglia/macrophages in which the MAPK cascades are highly activated after an ischemic injury [43]; [44]. In contrast, activation of ERK signaling pathway was critical for delayed neuroprotection [41]; [45]. We therefore investigated the effect of PF on the MAPK signaling pathways including JNK, p38 and ERK. We found that PF treatment potently restrained the activation of p38 MAPK and JNK, which were reported persistently activated during ischemia [43]; [44]. The expression of phosphorylated ERK was inhibited by ischemia in our results, whereas PF treatment promoted its activation. Therefore, MAPK pathway is involved in the protective effects of PF.

Compelling evidence indicates that apoptosis is crucially important in transient cerebral ischemia [46]. A variety of cell death signals in ischemia affect mitochondria, so the central role of mitochondria in apoptosis has been widely accepted [47]. The Bcl-2 proteins are a family of mitochondrial proteins involved in the response to apoptosis. Some of these proteins (such as bcl-2 and bcl-XL) are anti-apoptotic, while others (such as Bad, Bax or Bid) are pro-apoptotic [48]. Our results showed that ischemic insult significantly increased Bax expression but decreased Bcl-2 expression. The sensitivity of cells to apoptotic stimuli depends on the balance of pro- and anti-apoptotic bcl-2 proteins [49]. The pro- and antiapoptotic members of the Bcl-2 family control the release of cytochrome *c* and other factors from mitochondria. The release of cytochrome c from the mitochondria is particularly important in the induction of apoptosis. Once cytochrome c has been released into the cytosol, it is able to interact with Apaf-1 that leads to the recruitment of pro-caspase 9 into a multi-protein complex and results in the formation of apoptosome [50]. In our study, the increased levels of cytoplasmic cytochrome c were found in the ischemic rat brain which indicated more severe apoptosis in ischemia-injured rats. Importantly, we found that PF treatment for 14 days could efficaciously prevent the changes in these apoptosis-related proteins, thereby protecting against ischemia-induced neuronal loss in the rats.

The pro-inflammatory cytokine TNF plays a key role in a wide variety of physiological processes, including inflammation, proliferation and programmed cell death [51]. These pleiotropic biological effects of TNF result from its ability to initiate different intracellular signaling pathways. TNF binding to TNF receptor 1 (TNF-R1) leads to the recruitment of TNF-R associated death domain (TRADD), TNF-R associated factor 2 (TRAF2), and receptor interacting protein 1 (RIP1), forming complex I [51]. Signaling from complex I leads to NF-kB activation via activation of the IKK complex [52]. Signaling from complex I also activates the p38, ERK and JNK MAP kinases [53]. Whereas recruitment of FADD and procaspase-8 results in the formation of the cytosolic complex II, where caspase-8 is activated. Caspase-8 initiates the mitochondrial pathway by cleaving Bid to tBid, which induces mitochondrial permeabilization that results in the release of cytochrome c. This initiates an amplification loop that results in full-blown caspase activity and subsequent apoptosis [51]; [52]. Therefore, TNFα was used to activate MAPKs and NF-kB signal pathways in our in vitro study. The results revealed that PF could significantly protect against TNFα-induced hippocampal neuron damage, suggestting that MAPKs and NF-kB signal pathways were involved in PF-mediated neuroprotective effects.

In conclusion, our data demonstrate that PF produces a delayed protective effect on ischemic injury in the rats via inhibiting peripheral and cerebral inflammatory response. The MAPKs and NF-κB signaling pathways are involved in the protective effects of PF. The present study reveals that PF might be a potential neuroprotective agent for stroke.
